# Targeting BCL-2 in Cancer: Advances, Challenges, and Perspectives

**DOI:** 10.3390/cancers13061292

**Published:** 2021-03-14

**Authors:** Shirin Hafezi, Mohamed Rahmani

**Affiliations:** 1 Research Institute of Medical & Health Sciences, University of Sharjah, P.O. Box 27272 Sharjah, United Arab Emirates; shirin.a.hafezi@gmail.com; 2 Department of Basic Medical Sciences, College of Medicine, University of Sharjah, P.O. Box 27272 Sharjah, United Arab Emirates

**Keywords:** BCL-2, venetoclax, therapy, cancer, AML, CLL, resistance

## Abstract

**Simple Summary:**

Apoptosis, a programmed form of cell death, represents the main mechanism by which cells die. Such phenomenon is highly regulated by the BCL-2 family of proteins, which includes both pro-apoptotic and pro-survival proteins. The decision whether cells live or die is tightly controlled by a balance between these two classes of proteins. Notably, the pro-survival Bcl-2 proteins are frequently overexpressed in cancer cells dysregulating this balance in favor of survival and also rendering cells more resistant to therapeutic interventions. In this review, we outlined the most important steps in the development of targeting the BCL-2 survival proteins, which laid the ground for the discovery and the development of the selective BCL-2 inhibitor venetoclax as a therapeutic drug in hematological malignancies. The limitations and future directions are also discussed.

**Abstract:**

The major form of cell death in normal as well as malignant cells is apoptosis, which is a programmed process highly regulated by the BCL-2 family of proteins. This includes the antiapoptotic proteins (BCL-2, BCL-XL, MCL-1, BCLW, and BFL-1) and the proapoptotic proteins, which can be divided into two groups: the effectors (BAX, BAK, and BOK) and the BH3-only proteins (BIM, BAD, NOXA, PUMA, BID, BIK, HRK). Notably, the BCL-2 antiapoptotic proteins are often overexpressed in malignant cells. While this offers survival advantages to malignant cells and strengthens their drug resistance capacity, it also offers opportunities for novel targeted therapies that selectively kill such cells. This review provides a comprehensive overview of the extensive preclinical and clinical studies targeting BCL-2 proteins with various BCL-2 proteins inhibitors with emphasis on venetoclax as a single agent, as well as in combination with other therapeutic agents. This review also discusses recent advances, challenges focusing on drug resistance, and future perspectives for effective targeting the Bcl-2 family of proteins in cancer.

## 1. Introduction

One of the main steps of tumor initiation and progression is the evasion of apoptosis [1]. While cancer cells are fully equipped with apoptosis machinery, they develop various strategies to block the apoptosis process. Induction of apoptosis occurs through two main pathways: the intrinsic pathway generally activated by cellular stress (metabolism, oncogenes, DNA damage, nutrient deprivation, etc.) and the extrinsic pathway activated by death receptors (Tumor Necrosis Factors, Trail, Fas, etc.) [2]. Induction of these separated, but interconnected pathways, leads to activation of caspases, a class of cysteine proteases, which orchestrates the cleavage of vital cellular components leading to cellular death [3]. In the intrinsic pathway of apoptosis, caspases activation requires mitochondrial outer membrane permeabilization (MOMP), an event that leads to the release of cytochrome c from the mitochondrial intermembrane space into the cytosol [4]. These steps are tightly regulated by the BCL-2 family of proteins, which includes anti-apoptotic and pro-apoptotic proteins. The former includes BCL-2, BCL-XL, MCL-1, BCL-W, and BFL-1 also known as A1, whereas the latter includes effector proteins BAK, BAX, and BOK; and BH3 only proteins, which are grouped into sensitizers: BAD, NOXA, BMF, HRK, BIK; and activators: BIM, BID, and PUMA [5,6]. It is important to note that increasing evidence indicates that other BH-3 only proteins (e.g., NOXA) have the capacity to directly activate BAX and BAK, resulting in a less clear distinction between BH3-only sensitizer and activator proteins [7]. The ultimate decision whether a cell should live or die is controlled not only by the protein levels of the pro- and anti-apoptotic members, but also by the interplay between these two groups of proteins. In live cells, the proapoptotic BCL-2 proteins are largely sequestered by the anti-apoptotic members (BCL-2, BCL-XL, MCL-1, BCL-W, and BFL-1). However, under apoptotic insults, the BH3-only proteins play a central role in initiating apoptosis.

**Figure 1:**
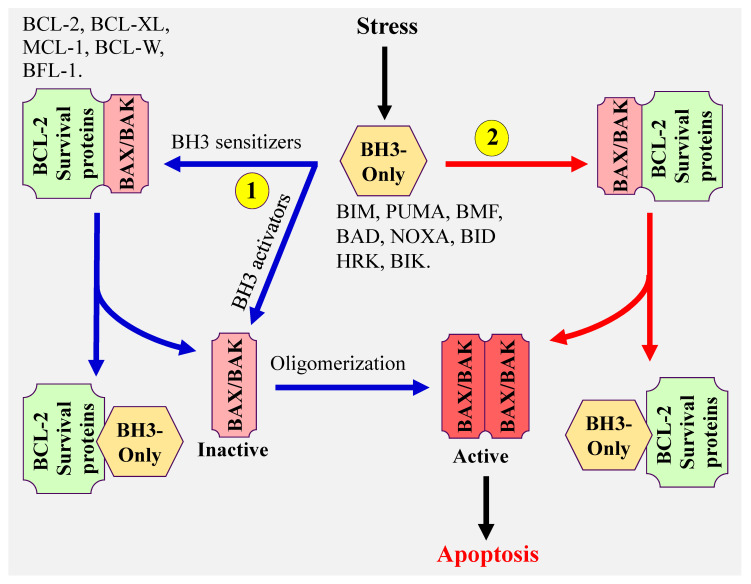
Model illustrating the mechanism(s) of mitochondrial apoptosis.

There are three proposed models that explain how BH3-only proteins induce apoptosis (Figure 1). (1) The direct model in which BH3-only activator proteins directly bind to and activate BAX and BAK, (2) the indirect model, also referred to as the displacement model. In this model, BH3 only sensitizer proteins, which do not have the capacity to effectively directly activate BAX and BAK, displace BAX and BAK from the antiapoptotic BCL-2 members (BCL-2, BCL-XL, MCL-1, BCL-W, and BFL-1), then BH3-only activator proteins activate the released BAX and BAK, and (3) the third model proposes the coexistence of both model 1 and 2. The common steps of all these models are the conformational change and oligomerization of BAX and BAK resulting in BAX/BAK pores formation and MOMP. These lead to the release of cytochrome c, activation of caspases [8,9] and ultimately induction of apoptosis. It is important to note that both BAX and BAK can also form homodimers, which assembles into higher order oligomers leading to the formation of pores, cytochrome c release, and apoptosis [10,11,12,13,14].

Accumulating evidences indicate that virtually all malignancies can be associated with apoptosis resistance; however, the mechanisms of this phenomenon differ from one tumor to another [15,16]. In this regard, overexpression of anti-apoptotic BCL-2 members (e.g., BCL-2, BCL-XL, MCL-1) is quite frequent in newly diagnosed cancer as well as after developing resistance to therapies [17,18]. Conversely, loss or downregulation of the pro-apoptotic BCL-2 members have been observed in many tumor types [6]. Additionally, the ability to activate the BH3 only proteins PUMA and NOXA is often impaired due to p53 deficiency in many cancer cells [19]. Another mechanism of apoptotic evasion involves epigenetic aberration. In this regard, silencing of BIM and PUMA through promoter hypermethylation is frequently observed in Burkitt lymphomas and chronic myeloid leukemia (CML) [20,21]. These considerations have prompted extensive efforts aiming at targeting the pro-survival BCL-2 members.

## 2. Nonspecific BCL-2 Inhibitor

One of the first compounds found to inhibit BCL-2 functions is gossypol, a natural polyphenol isolated from cotton seeds and roots. It is well recognized that gossypol exerts its anticancer activity through a variety of mechanisms including inhibition of the pro-survival BCL-2 proteins. It can also induce the pro-apoptotic BH3-only proteins NOXA and PUMA through a p53-independent mechanism [22]. Like other BCL-2 inhibitors, gossypol disrupts the equilibrium between the anti- and pro-apoptotic BCL-2 members leading to BAX/BAK activation and apoptosis. However, gossypol can also induce cell death independently of BAX and BAK highlighting the pleotropic nature and suggesting an alternative mechanism of cell death mediated by this agent [23]. In this regard, multiple studies have found that gossypol effectively disrupts many cell signaling pathways [24]. For example, Moon et al. [25] have reported that gossypol suppresses NF-kB activity and downregulates many NF-kB-regulated genes including cIAP-1/2 (cellular inhibitor of apoptosis proteins 1 and 2), and XIAP (X-linked IAP). Gossypol can also disrupt the binding of MDM2 protein to VEGF mRNA 3′-UTR leading to MDM2 self-ubiquitination and proteasomal degradation, and a diminution of VEGF m-RNA translation [26].

During the last two decades, gossypol has undergone extensive clinical evaluation. For example, a phase I/II studies using 23 patients with progressive castrate-resistant prostate cancer have revealed a significant toxicity and a lack of activity of gossypol as a single agent [27].

## 3. Selective Targeting of BCL-2 Pro-Survival Members Using BH3 Mimetics

**Figure 2:**
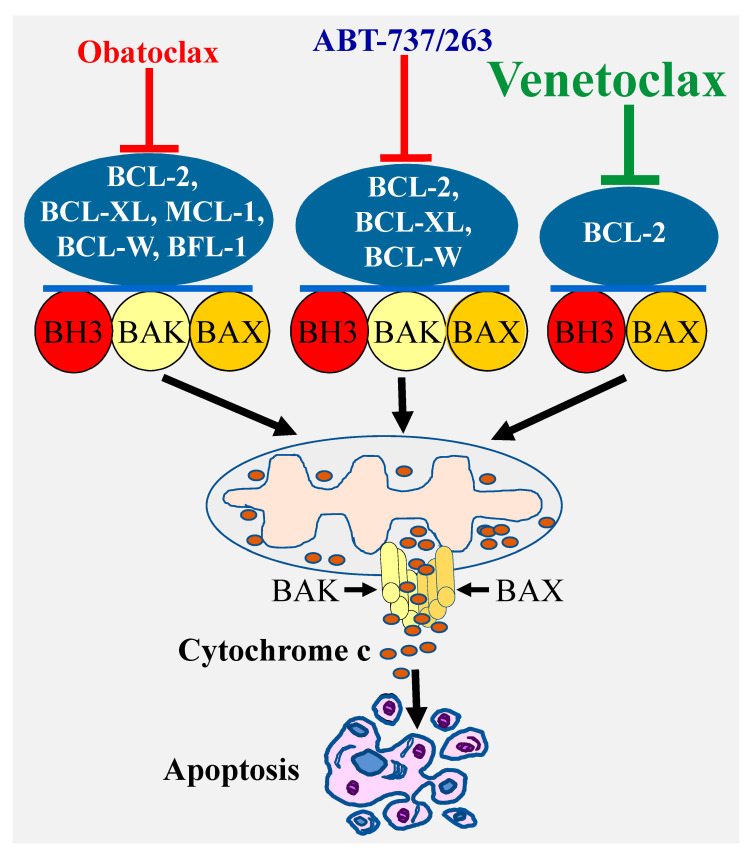
Specificity of various types of BCl-2 inhibitors and induction of apoptosis. Targeting the Bcl-2 pro-survival proteins by various inhibitors with different specificity (ABT-737/ABT-263, obatoclax, or venetoclax) leads to BAX/BAK activation and pores formation through which cytochrome c is released into the cytosol culminating in apoptosis induction.

A highly effective approach for selective targeting of BCL-2 pro-survival proteins has been focusing on developing BH3 mimetics, which directly bind with high affinity to the hydrophobic grooves of the pro-survival proteins. In this regard, the deep understanding of the BCL-2 proteins biology coupled with the remarkable advances in the structure-based drug design have led to the discovery of a number of BCL-2 inhibitors. The first highly selective BCL-2 inhibitors ABT-737 was discovered using nuclear magnetic resonance structure-based design with the BH3 region of BAD [28]. This compound and its clinical derivative navitoclax (ABT-263) target BCL-2, BCL-XL, and BCL-W [29] (Figure 2). Another important advance was the discovery of a less selective Bcl-2 inhibitor obatoclax (GX15-070), which targets all the BCL-2 pro-survival members BCL-2, BCL-XL, MCL-1, and BCL-W [30]. These discoveries were subsequently followed by the development of venetoclax (ABT-199), which selectively targets BCL-2 protein [31] (Figure 2).

### 3.1. ABT-737 and ABT-263 (Navitoclax)

While ABT-737 has shown a marked activity in preclinical studies in various types of cancer, its poor pharmacokinetic and solubility were major challenges for its development. Its clinical derivative navitoclax (ABT-263) was also highly active in preclinical studies. In addition, phase I clinical trials conducted in patients with non-small cell lung cancer (NSCLC) or lymphoid malignancies [32,33] have shown that navitoclax is safe and well tolerated; however a dose-dependent thrombocytopenia, a consequence of BCL-XL inhibition [34], was a major adverse effect. Such a problem has prevented the use of sufficient navitoclax doses for effective cancer cell killing. These considerations have been corroborated by a phase II study in advanced and recurrent small cell lung cancer (SCLC), which has shown only a limited navitoclax single-agent activity [35]. In addition, navitoclax enhances the activity of multiple therapeutic agents in patients with solid tumors as well as hematological malignancies, suggesting that this agent might be useful for combination therapy at least in some tumor types [36,37,38,39]. In this regard, a very recent single arm phase IB study has shown a promising activity of combined treatment with navitoclax and EGFR inhibitor osimertinib in patients with advanced EGFR-mutant NSCLC [40].

In addition to navitoclax, a recent study has described a novel dual BCL-2/BCL-XL inhibitor AZD4320, which appears to be highly active in acute myeloid leukemia (AML) preclinical models with reduced thrombocytopenia [41]. However, the development of AZD4320 has been hampered due to its cardiovascular toxicity observed in preclinical studies [42]. To circumvent this problem, Patterson et al. have chemically conjugated AZD4320 to PEGylated poly-lysine dendrimer [42]. Notably, the resulting agent referred to as AZD0466 showed significant preclinical activity and improved tolerability compared to AZD4320 in a human leukemia xenograft mouse model [42].

Collectively, these considerations strongly suggest that improved drug design combined with patient selections and dose optimization might lead to effective anticancer treatment involving this class of agents.

### 3.2. Obatoclax (GX15-070)

Obatoclax (GX15-070) is a hydrophobic small molecule that binds with high affinity to BH3 binding groove of all the pro-survival BCL-2 members [30,43] unlike ABT-737 and navitoclax which spare MCL-1, and BFL-1. Numerous preclinical studies have shown that obatoclax exhibits a potent antitumor activity in various types of cancer both in vitro and in vivo [30,44,45,46]. Mechanistically, obatoclax induces apoptosis through activation of BAX/BAK following their release from the pro-survival BCL-2 members [43,45].

Obatoclax has undergone extensive clinical evaluation in various hematological malignancies as well as solid tumors, however, significant toxicity and modest or lack of activity have limited its development for cancer therapy. Initial obatoclax phase I clinical trial was conducted in 44 patients with advanced hematological malignancies [47]. This included patients with chronic lymphocytic leukeamia (CLL, 9%), refractory acute myeloid leukemia (AML, 57%), acute lymphoblatic leukemia (ALL, 2%) and myelodysplasia (MDS, 32%). The clinical responses were modest, only 3 of 14 MDS patients showed improvement and one patient with refractory AML t(9;11) translocation achieved a complete remission that lasted 8 months [47]. In addition, grade 1/2 central nervous system side effects were observed. A second phase I/II clinical trial examining obatoclax in 18 untreated AML patients older than 70 years has led to similar results [48].

The adverse effects of obatoclax may result from many factors including (a) inhibition of all antiapoptotic BCL-2 members, which might be toxic to many normal cells. In this regard, MCL-1 is known to play a critical role in stem cell survival particularly hematopoietic stem cells [49,50]. BCL-XL is also required for megakaryocytes survival, and disabling BCL-XL leads to a decrease in platelets production, which can potentially cause thrombocytopenia [33,34], and (b) lack of obatoclax specificity as obatoclax can also dysregulate the activity of many survival factors. For example, obatoclax decreases cyclin D1 and Cdk4/6 expression levels [46,51] and inhibits NF-kB [52].

In conclusion, targeting all pro-survival Bcl-2 proteins might not offer sufficient selectivity for cancer cells to provide a therapeutic outcome.

### 3.3. Venetoclax (ABT-199)

While the functional redundancy among BCL-2, BCL-XL, and Mcl-1 suggests that selective targeting of BCL-2 protein may not lead to extensive cancer cell death, the lack of clinical activity and or excessive toxicity of pan-BCL-2 or dual BCL-2/BCL-XL inhibitors have prompted the development of highly selective inhibitors for individual BCL-2 members. Venetoclax, also referred to as ABT-199, was the first highly selective BCL-2 inhibitor to be discovered [31]. It is a BH3-mimetic that binds to the BH3-binding groove of BCL-2 protein with high affinity. Surprisingly, venetoclax has showed a rapid and striking activity in many cancer models. These observations support the notion that despite the functional redundancy among the antiapoptotic BCL-2 members, some cancer types are particularly dependent on BCL-2 for their survival.

## 4. Venetoclax in the Treatment of CLL

It is well established that resistance against radiation, chemotherapy, and genotoxic stress is highly associated with overexpression of BCL-2 in many malignancies [53,54]. This occurs through a variety of mechanisms. In CLL, overexpression of BCL-2 has been linked to the loss of the tumor suppressors microRNAs miR-15 and miR-16, two major negative regulators of BCL-2 expression, due to 13q deletion. Notably, expression of miR-15/16 was downregulated in approximately 70% of CLL patients [55,56]. It is important to note that other mechanisms can lead to miR-15 and miR-16 loss in CLL including epigenetic mechanisms [56,57,58].

Importantly, the profound induction of apoptosis by venetoclax in cancer was associated with much less thrombocytopenia compared to agents that inhibit BCL-XL such as navitoclax or obatoclax [31]. These findings have prompted a multicenter phase I study of venetoclax in patients with relapsed or refractory CLL or small lymphocytic lymphoma (SLL) [59]. While initial dose-escalation schedule in patients with CLL has been challenged by significant tumor lysis syndrome, which occurred in 3 out of 56 patients, adjustment of dose-escalation schedule has successfully prevented the occurrence of this form of toxicity [59]. This trial has demonstrated an impressive activity of venetoclax in CLL. In fact, 92 of 116 patients (79%), the majority of which have adverse prognosis, showed clinical response. Among these, 20% achieved complete remission. Notably, daily administration of 400 mg venetoclax has led to 15-month progression-free survival in 69% of patients. Consistent with these findings, studies conducted in primary CLL cells isolated from 33 patients revealed that venetoclax induced a rapid (few hours) and profound mitochondrial apoptosis [60].

A phase II, single-arm multicenter study, with venetoclax monotherapy in patients with relapsed or refractory CLL with 17p deletion has led to an overall response rate of 79.4% [61]. Importantly, neutropenia (40%), infection (20%), anaemia (18%), and thrombocytopenia (15%) were the most common grade 3–4 adverse events [61].

Together, these studies have led to the venetoclax approval by US Food and Drug Administration (FDA) and European Medicines Agency (EMA) for the treatment of patients with 17p deleted relapsed/refractory CLL, a population with very poor prognosis [62].

Despite the impressive activity of venetoclax as a single agent in CLL in the above trials, the observations that the estimated 24 months progression-free survival in a phase II study was 54% [59] and that complete remission occurred only in 20% of patients [59,63] indicate a significant resistance against this agent in the majority of patients. These findings suggest that combination with novel targeted agents might be an effective strategy to overcome resistance to venetoclax. In this regard, multiple studies have recently assessed the clinical efficacy of venetoclax in combination with standard therapy. For example, in a phase Ib trial in 49 relapsed or refractory CLL/SLL patients, combined treatment with venetoclax and the anti-CD20 monoclonal antibody rituximab achieved a complete response in 50% patients [64]. In a more recent phase III study in patients with relapsed or refractory CLL, venetoclax-rituximab combination therapy achieved a significantly higher percentage of progression-free survival compared to a standard chemoimmunotherapy with bendamustine and rituximab [65]. Another important finding in these studies is that venetoclax/rituximab was active across all clinical and biologic subgroups regardless of the 17p deletion status. Specifically, the 24 months progression-free survival achieved by this regimen was 81.5% in patients with 17p deletion versus 85.9% in patients without such chromosomal translocation. Significantly, combined treatment with veneteclax and another anti-CD20 monoclonal antibody obinutuzumab was highly active in patients with previously untreated CLL and coexisting conditions in a phase III trial [66]. While after two years of treatment, the progression-free survival was 88.2% for the venetoclax-obinutuzumab group, this was only 64.1% for patients treated with chlorambucil-obinutuzumab. Similar to venetoclax/rituximab, venetoclax/obinutuzumab benefit was observed in patients with various genetic backgrounds such as TP53 deletion and/or mutation, or unmutated immunoglobulin heavy-chain genes. Noteworthy, the side effects of such regimen (e.g., grade 3 or 4 neutropenia and infections) were comparable to those caused by chlorambucil-obinutuzumab.

## 5. Venetoclax in the Treatment of AML

While a large number of agents have failed in various stages of clinical trials in AML over several decades, recently, FDA approved the use of venetoclax in combination with the hypomethylating agents azacitidine or decitabine or low-dose of cytarabine as frontline therapies in patients over 70 years of ages or who cannot tolerate aggressive chemotherapeutic treatment [67,68].

Initial preclinical studies have shown that venetoclax exhibited impressive antitumor activities in AML cells displaying diverse genetic backgrounds including MLL-rearrangement or IDH1/2, or NPM1 mutations [69,70,71,72]. Conversely, AML cells with some genetic backgrounds such as mutations in FLT3, p53, or Ras, have been shown to be quite resistant to this agent [69,70,71,72]. Importantly, venetoclax was found to have minimal effect on platelet levels raising the possibility that this agent may achieve a clinical outcome at least in some patients with AML. In fact, in a phase II trial, venetoclax showed a significant single agent activity in AML [73]. This study was conducted in 32 AML patients with high-risk relapsed/refractory or unfit for intensive chemotherapy, and with a median age of 71 years. The overall response rate was 19% [73]. Interestingly, patients with IDH1/2 mutations showed the highest response [73]. However, unlike CLL, AML patients who initially responded to venetoclax have rapidly (2.5 months) developed resistance to this agent [74]. In addition, although venetoclax showed evidence of activity in AML, this was lower compared to that obtained with hypomethylating agents in elderly AML patients [75].

The limited anti-leukemic activity of venetoclax as a single agent, and the rapid development of resistance have prompted multiple clinical trials involving combination with chemotherapeutic agents. In fact, combined treatment with venetoclax and hypomethylating agents-azacytidine and/or decitabine have led to impressive activity in 60 years or older AML patients. Such treatments were particularly effective in newly diagnosed patients and also, to a lesser extent, in relapsed or refractory patients [76,77,78,79]. Another very important phase Ib/II study demonstrated that combinatorial treatment involving venetoclax and the IDH1 inhibitor ivosidenib with or without azacytidine, is well tolerated and highly effective in IDH1-mutant AML patients [80].

On November 2018, the FDA granted accelerated approval for venetoclax in combination with the hypomethylating agents azacitidine or decitabine or low-dose of cytarabine as a front-line therapy in AML patients who cannot tolerate aggressive chemotherapy [81]. It is important to note that despite the impressive activity of combined treatment with venetoclax and hypomethylating agents in AML, such regiment failed to achieve clinical response in approximately 39% of patients and most of patients who initially achieved remission develop resistance in less than two years [78,79].

## 6. Venetoclax in the Treatment of Other Hematological Malignancies

In addition to CLL and AML, venetoclax has also been evaluated in other hematological malignancies including lymphomas and multiple myeloma.

A recent phase I clinical trial has examined venetoclax in relapsed or refractory patients with diverse non-Hodgkin lymphoma (NHL) subtypes including mantle cell lymphoma (MCL), follicular lymphoma (FL), diffuse large B-cell lymphoma (DLBCL), DLBCL arising from chronic lymphocytic leukemia (Richter transformation), Waldenström macroglobulinemia, and marginal zone lymphoma [82]. In this study, venetoclax was well tolerated with the highest overall response rate observed in MCL (44%) followed by FL (38%) and DLBCL (18%). The progression-free survival was 14, 11, and 1 months, respectively. Another recent phase I/II trial in relapsed/refractory NHL has led to similar results [83]. The median progression-free survival was two months and the overall survival was 4.5 months.

Venetoclax has also been tested in combination with bortezomib and dexamethasone in patients with relapsed/refractory multiple myeloma in a phase III clinical trial [84]. However, despite the superior clinical activity of regimens involving venetoclax compared to placebo, a substantial increase in mortality was observed in the venetoclax group, mostly due to high infections rate.

Together, these findings suggest that patient selection might improve the therapeutic activity of venetoclax as single agent or in combination therapy.

## 7. Resistance of Cancer Cells to Venetoclax

Although venetoclax has demonstrated impressive initial clinical activity in various hematological malignancies; intrinsic or acquired drug resistance represents a major obstacle for achieving effective and durable therapy with this agent.

While it is clear that many malignant cells depend on BCL-2 for their survival, the molecular basis for this dependency remains poorly understood. A number of studies have linked venetoclax sensitivity to the abundance of BCL-2 protein in cells. A high expression of BCL-2 seems to be associated with high sensitivity to venetoclax in many tumor models [31,54,56,85,86]. However, some malignant cells display high BCL-2 protein level but exhibit significant resistance to venetoclax [31,72,86]. Conversely, some tumors cells express low level of BCL-2 protein, but they are highly sensitive to venetoclax [85,86]. These observations clearly demonstrate that BCL-2 protein level is not the sole determinant of cancer cells sensitivity to venetoclax. Significantly, studies from our group and others have demonstrated that MCL-1 or BCL-XL proteins also play a critical role in antagonizing venetoclax activity [72,87].

Another factor that plays a critical role in determining mitochondrial apoptosis is the interplay between the antiapoptotic and the proapoptotic BCL-2 members. In this context, multiple studies have suggested that the BH3-only protein BIM may play an important role in venetoclax activity. For example, sequestration of BIM by MCL-1 has been identified as a mechanism of intrinsic venetoclax resistance [88]. However, numerous other studies using siRNA or CRISPR system have shown that BIM or other BH3-only proteins such as PUMA, and NOXA are dispensable for BCL-2 inhibitors apoptotic activity [72,89,90]. A common finding in all these studies is that BAX and BAK play a critical role in BCL-2 inhibitors-mediated cell killing. Together these observations support a model in which venetoclax induces apoptosis through the displacement model in which venetoclax efficiently displaces BAX from BCL-2 leading to its activation, oligomerization with BAK, and initiation of the apoptotic cascade. However, these findings do not exclude the direct contribution of BH3-only protein in BAX and or BAK activation. They rather suggest that BAX displacement from BCL-2 by venetoclax is sufficient to induce activation of BAX which, in cooperation with BAK, effectively induces apoptosis without the requirement of other activators.

Another important, but less understood, aspect of cancer cells sensitivity to venetoclax is their genetic backgrounds. Cancer cells baring some types of genetic aberrations such as chromosomal rearrangement [71], IDH1/2, or WT1 point mutations [70,91] are exquisitely sensitive to venetoclax. The molecular basis of this sensitivity has been linked to high level of BCL-2 in these cells; however, additional factors might be determinant in this phenomenon. In contrast, several aberrations have been associated with resistance to venetoclax, although in some cases no direct links between these aberrations and BCL-2 expression have been demonstrated. These include overexpression of MCL-1 or BCL-XL, mutations in JAK2, Ras, p53, FLT3 (FLT3-ITD), β2-microglobulin, and complex cytogenetics [86,87,92]. Several studies have also suggested that BCL-2/BIM ratio might predict for AML sensitivity to venetoclax [88,93]. However, the mechanism of cancer cells sensitivity to venetoclax would likely be multifactorial and context dependent. In fact, recent studies have identified many mutations in BCL-2 coding sequence some of which plays critical roles in resistance to venetoclax.

## 8. Venetoclax Resistance Mediated by BCL-2 Mutations

Despite the impressive initial clinical activity of venetoclax as a single agent in various hematological malignancies particularly in CLL, acquired resistance is a major problem for most patients [59,63] as is the case for many targeted agents.

Recent studies have identified two key BCL-2 mutations in venetoclax-resistant CLL patients. The most frequent mutation involves a substitution of glycine with valine at position 101 (G101V), and a less prominent mutation consists of a substitution of aspartic acid with tyrosine at position 103 (D103Y) [94,95,96,97]. These mutations were not detected in patients before treatment, they were first detected only after 19 months of venetoclax treatment. Importantly, the development of these mutations was highly associated with the emergence of venetoclax resistance. In vitro studies have revealed that G101V and D1013Y mutations abrogates venetoclax activity by dramatically reducing its affinity for BCL-2 [96,97].

Another study by Correia et al. [98] has described various BCL-2 mutations in follicular lymphoma, some of which occur in 12% of patients at diagnosis, but the majority of these mutations occur at transformation raising the possibility that these mutations may increase the risk of follicular lymphoma transformation. Of note follicular lymphoma is an indolent malignancy highly associated with overexpression of BCL-2 as a consequence of t(14;18) chromosomal translocation, but frequently undergoes transformation to a more aggressive type of lymphoma, particularly diffuse large B-cell lymphoma [99]. Functional analysis revealed that some of these mutations (BCL-2 G33R or A43T) markedly increased BCL-2 ability to bind the proapoptotic BIM and PUMA, thereby protecting cells against apoptosis [98].

Cancer cells might involve other mechanisms of resistance to venetoclax. For example, Fresquet et al., [100] have reported a missense mutation in the C-terminal transmembrane domain of BAX (G179E) in a human lymphoma cell line rendered resistant to venetoclax in vitro. Such mutation diminishes BAX binding ability to the mitochondrial membrane and thereby prevents BAX-mediated apoptosis [100]. However, this was not observed in mouse-derived lymphoma cells rendered resistant to venetoclax through the same process. Instead, these cells exhibited mutations (F101C and F101L) in the BH3 domain of BCL-2 [100], which abrogates venetoclax binding to the BCL-2 BH3 domain and ultimately prevents mitochondrial apoptosis,

Together, these observations argue that venetoclax-resistance may occur through diverse mechanisms that stem from various types of mutations affecting both anti-apoptotic and proapoptotic BCL-2 members and might be cell type-specific.

## 9. Strategies to Overcome Venetoclax Resistance and Perspectives

While preclinical studies using combined treatment with venetoclax and MCL-1 inhibitors in hematological malignancies has generated a substantial enthusiasm for overcoming venetoclax resistance [101,102], clinical evaluations appear to reveal excessive toxicity of such regimens [103]. Accumulating evidence suggest that rational combination strategies involving venetoclax and other standard therapy or novel targeted agents might lead to effective treatments; however, the emergence of BCL-2 mutations that cause resistance to venetoclax highlights the need for developing mutation-specific BCL-2 inhibitors to overcome such drug resistance. A recently emerged antitumor approach is the direct activation of the proapoptotic effector protein BAX by a new class of BAX agonists [104,105,106]. Such an approach may provide a therapeutic benefit at least in some cancer setting. Developing additional Bcl-2 inhibitors with different affinity for other pro-survival BCL-2 members might also be useful in cancer with some genetic background. In this regard, a recent study has described a novel orally bioavailable BCL-2 inhibitor referred to as BCL201 (S55746), which selectively binds to the hydrophobic groove of BCL-2, poorly binds to BCL-XL, and spares the other BCL-2 survival proteins MCL-1 and BFL-1 [107]. Importantly, S55746 exhibited profound antitumor activities in various hematological malignancies both in vitro and in vivo models [107].

Nevertheless, despite the remarkable advances in targeting BCL-2 members for cancer therapy, a better understanding of mechanisms underlying cancer cells resistance to venetoclax as a single agent as well as in combination with other agents is needed for developing safe, effective, and durable treatments. Patient selections and development of biomarkers determinant of patient response might also be critical for improving the therapeutic outcomes of BCL-2 inhibitors in diverse types of cancer.

## 10. Conclusions

While venetoclax has demonstrated impressive activity in CLL and AML either as single agent or in combination with hypomethylating agents or low-dose cytarabine (LDAC), the development of drug resistance in these diseases and the lack of activity in other cancer types particularly in solid tumors have limited its therapeutic use. Given the favorable safety profile of venetoclax, a greater understanding of the mechanisms of primary as well as acquired resistance to this agent is needed to improve or extent venetoclax-based therapies, and potentially other Bcl-2 inhibitors, to various tumor types including solid tumors.

## References

[1] Hanahan D., Weinberg R.A. (2011). Hallmarks of cancer: The next generation. Cell.

[2] Testa U., Riccioni R. (2007). Deregulation of apoptosis in acute myeloid leukemia. Haematologica.

[3] Adams J.M., Cory S. (2018). The BCL-2 arbiters of apoptosis and their growing role as cancer targets. Cell Death Differ..

[4] Kalkavan H., Green D.R. (2017). MOMP, cell suicide as a BCL-2 family business. Cell Death Differ..

[5] Bhola P.D., Letai A. (2016). Mitochondria-Judges and Executioners of Cell Death Sentences. Mol. Cell.

[6] Campbell K.J., Tait S.W.G. (2018). Targeting BCL-2 regulated apoptosis in cancer. Open Biol.

[7] Luo X., O’Neill K.L., Huang K. (2020). The third model of Bax/Bak activation: A Bcl-2 family feud finally resolved?. F1000Research.

[8] Kuwana T., Bouchier-Hayes L., Chipuk J.E., Bonzon C., Sullivan B.A., Green D.R., Newmeyer D.D. (2005). BH3 domains of BH3-only proteins differentially regulate Bax-mediated mitochondrial membrane permeabilization both directly and indirectly. Mol. Cell.

[9] Singh R., Letai A., Sarosiek K. (2019). Regulation of apoptosis in health and disease: The balancing act of BCL-2 family proteins. Nat. Rev. Mol. Cell Biol..

[10] Schlesinger P.H., Saito M. (2006). The Bax pore in liposomes, Biophysics. Cell Death Differ..

[11] Qian S., Wang W., Yang L., Huang H.W. (2008). Structure of transmembrane pore induced by Bax-derived peptide: Evidence for lipidic pores. Proc. Natl. Acad. Sci. USA.

[12] Zhang M., Zheng J., Nussinov R., Ma B. (2017). Release of Cytochrome C from Bax Pores at the Mitochondrial Membrane. Sci. Rep..

[13] Westphal D., Dewson G., Menard M., Frederick P., Iyer S., Bartolo R., Gibson L., Czabotar P.E., Smith B.J., Adams J.M. (2014). Apoptotic pore formation is associated with in-plane insertion of Bak or Bax central helices into the mitochondrial outer membrane. Proc. Natl. Acad. Sci. USA.

[14] Uren R.T., O’Hely M., Iyer S., Bartolo R., Shi M.X., Brouwer J.M., Alsop A.E., Dewson G., Kluck R.M. (2017). Disordered clusters of Bak dimers rupture mitochondria during apoptosis. eLife.

[15] Cassier P.A., Castets M., Belhabri A., Vey N. (2017). Targeting apoptosis in acute myeloid leukaemia. Br. J. Cancer.

[16] Hainaut P., Pfeifer G.P. (2016). Somatic TP53 Mutations in the Era of Genome Sequencing. Cold Spring Harb. Perspect. Med..

[17] Kaufmann S.H., Karp J.E., Svingen P.A., Krajewski S., Burke P.J., Gore S.D., Reed J.C. (1998). Elevated expression of the apoptotic regulator Mcl-1 at the time of leukemic relapse. Blood.

[18] Um H.D. (2016). Bcl-2 family proteins as regulators of cancer cell invasion and metastasis: A review focusing on mitochondrial respiration and reactive oxygen species. Oncotarget.

[19] Villunger A., Michalak E.M., Coultas L., Mullauer F., Bock G., Ausserlechner M.J., Adams J.M., Strasser A. (2003). p53- and drug-induced apoptotic responses mediated by BH3-only proteins puma and noxa. Science.

[20] San Jose-Eneriz E., Agirre X., Jimenez-Velasco A., Cordeu L., Martin V., Arqueros V., Garate L., Fresquet V., Cervantes F., Martinez-Climent J.A. (2009). Epigenetic down-regulation of BIM expression is associated with reduced optimal responses to imatinib treatment in chronic myeloid leukaemia. Eur. J. Cancer.

[21] Garrison S.P., Jeffers J.R., Yang C., Nilsson J.A., Hall M.A., Rehg J.E., Yue W., Yu J., Zhang L., Onciu M. (2008). Selection against PUMA gene expression in Myc-driven B-cell lymphomagenesis. Mol. Cell Biol..

[22] Meng Y., Tang W., Dai Y., Wu X., Liu M., Ji Q., Ji M., Pienta K., Lawrence T., Xu L. (2008). Natural BH3 mimetic (-)-gossypol chemosensitizes human prostate cancer via Bcl-xL inhibition accompanied by increase of Puma and Noxa. Mol. Cancer Ther..

[23] Vogler M., Weber K., Dinsdale D., Schmitz I., Schulze-Osthoff K., Dyer M.J., Cohen G.M. (2009). Different forms of cell death induced by putative BCL2 inhibitors. Cell Death Differ..

[24] Keshmiri-Neghab H., Goliaei B. (2013). Therapeutic potential of gossypol: An overview. Pharm. Biol..

[25] Moon D.O., Kim M.O., Lee J.D., Kim G.Y. (2008). Gossypol suppresses NF-kappaB activity and NF-kappaB-related gene expression in human leukemia U937 cells. Cancer Lett..

[26] Xiong J., Li J., Yang Q., Wang J., Su T., Zhou S. (2017). Gossypol has anti-cancer effects by dual-targeting MDM2 and VEGF in human breast cancer. Breast. Cancer Res..

[27] Liu G., Kelly W.K., Wilding G., Leopold L., Brill K., Somer B. (2009). An open-label, multicenter, phase I/II study of single-agent AT-101 in men with castrate-resistant prostate cancer. Clin. Cancer Res..

[28] Oltersdorf T., Elmore S.W., Shoemaker A.R., Armstrong R.C., Augeri D.J., Belli B.A., Bruncko M., Deckwerth T.L., Dinges J., Hajduk P.J. (2005). An inhibitor of Bcl-2 family proteins induces regression of solid tumours. Nature.

[29] Park C.M., Bruncko M., Adickes J., Bauch J., Ding H., Kunzer A., Marsh K.C., Nimmer P., Shoemaker A.R., Song X. (2008). Discovery of an orally bioavailable small molecule inhibitor of prosurvival B-cell lymphoma 2 proteins. J. Med. Chem..

[30] Trudel S., Li Z.H., Rauw J., Tiedemann R.E., Wen X.Y., Stewart A.K. (2007). Preclinical studies of the pan-Bcl inhibitor obatoclax (GX015-070) in multiple myeloma. Blood.

[31] Souers A.J., Leverson J.D., Boghaert E.R., Ackler S.L., Catron N.D., Chen J., Dayton B.D., Ding H., Enschede S.H., Fairbrother W.J. (2013). ABT-199, a potent and selective BCL-2 inhibitor, achieves antitumor activity while sparing platelets. Nat. Med..

[32] Gandhi L., Camidge D.R., Ribeiro de Oliveira M., Bonomi P., Gandara D., Khaira D., Hann C.L., McKeegan E.M., Litvinovich E., Hemken P.M. (2011). Phase I study of Navitoclax (ABT-263), a novel Bcl-2 family inhibitor, in patients with small-cell lung cancer and other solid tumors. J. Clin. Oncol..

[33] Wilson W.H., O'Connor O.A., Czuczman M.S., LaCasce A.S., Gerecitano J.F., Leonard J.P., Tulpule A., Dunleavy K., Xiong H., Chiu Y.L. (2010). Navitoclax, a targeted high-affinity inhibitor of BCL-2, in lymphoid malignancies: A phase 1 dose-escalation study of safety, pharmacokinetics, pharmacodynamics, and antitumour activity. Lancet Oncol..

[34] Mason K.D., Carpinelli M.R., Fletcher J.I., Collinge J.E., Hilton A.A., Ellis S., Kelly P.N., Ekert P.G., Metcalf D., Roberts A.W. (2007). Programmed anuclear cell death delimits platelet life span. Cell.

[35] Rudin C.M., Hann C.L., Garon E.B., Ribeiro de Oliveira M., Bonomi P.D., Camidge D.R., Chu Q., Giaccone G., Khaira D., Ramalingam S.S. (2018). Phase II study of single-agent navitoclax (ABT-263) and biomarker correlates in patients with relapsed small cell lung cancer. Clin. Cancer Res..

[36] Cleary J.M., Lima C.M., Hurwitz H.I., Montero A.J., Franklin C., Yang J., Graham A., Busman T., Mabry M., Holen K. (2014). A phase I clinical trial of navitoclax, a targeted high-affinity Bcl-2 family inhibitor, in combination with gemcitabine in patients with solid tumors. Investig. New Drugs.

[37] Roberts A.W., Advani R.H., Kahl B.S., Persky D., Sweetenham J.W., Carney D.A., Yang J., Busman T.B., Enschede S.H., Humerickhouse R.A. (2015). Phase 1 study of the safety, pharmacokinetics, and antitumour activity of the BCL2 inhibitor navitoclax in combination with rituximab in patients with relapsed or refractory CD20+ lymphoid malignancies. Br. J. Haematol..

[38] Tolcher A.W., LoRusso P., Arzt J., Busman T.A., Lian G., Rudersdorf N.S., Vanderwal C.A., Kirschbrown W., Holen K.D., Rosen L.S. (2015). Safety, efficacy, and pharmacokinetics of navitoclax (ABT-263) in combination with erlotinib in patients with advanced solid tumors. Cancer Chemother. Pharmacol..

[39] Vlahovic G., Karantza V., Wang D., Cosgrove D., Rudersdorf N., Yang J., Xiong H., Busman T., Mabry M. (2014). A phase I safety and pharmacokinetic study of ABT-263 in combination with carboplatin/paclitaxel in the treatment of patients with solid tumors. Investig. New Drugs.

[40] Bertino E.M., Gentzler R.D., Clifford S.E., Kolesar J.M., Muzikansky A., Haura E.B., Piotrowska Z., Camidge D.R., Stinchcombe T.E., Hann C.L. (2020). Phase IB study of osimertinib in combination with navitoclax in EGFR-mutant NSCLC following resistance to initial EGFR therapy (ETCTN 9903). Clin. Cancer Res..

[41] Balachander S.B., Criscione S.W., Byth K.F., Cidado J., Adam A., Lewis P., Macintyre T., Wen S., Lawson D., Burke K. (2020). AZD4320, A Dual Inhibitor of Bcl-2 and Bcl-xL, Induces Tumor Regression in Hematologic Cancer Models without Dose-limiting Thrombocytopenia. Clin. Cancer Res..

[42] Patterson C.M., Balachander S.B., Grant I., Pop-Damkov P., Kelly B., McCoull W., Parker J., Giannis M., Hill K.J., Gibbons F.D. (2021). Design and optimisation of dendrimer-conjugated Bcl-2/xL inhibitor, AZD0466, with improved therapeutic index for cancer therapy. Commun. Biol..

[43] Nguyen M., Marcellus R.C., Roulston A., Watson M., Serfass L., Murthy Madiraju S.R., Goulet D., Viallet J., Belec L., Billot X. (2007). Small molecule obatoclax (GX15-070) antagonizes MCL-1 and overcomes MCL-1-mediated resistance to apoptosis. Proc. Natl. Acad. Sci. USA.

[44] Campas C., Cosialls A.M., Barragan M., Iglesias-Serret D., Santidrian A.F., Coll-Mulet L., de Frias M., Domingo A., Pons G., Gil J. (2006). Bcl-2 inhibitors induce apoptosis in chronic lymphocytic leukemia cells. Exp. Hematol..

[45] Rahmani M., Aust M.M., Attkisson E., Williams D.C., Ferreira-Gonzalez A., Grant S. (2012). Inhibition of Bcl-2 antiapoptotic members by obatoclax potently enhances sorafenib-induced apoptosis in human myeloid leukemia cells through a Bim-dependent process. Blood.

[46] Or C.R., Chang Y., Lin W.C., Lee W.C., Su H.L., Cheung M.W., Huang C.P., Ho C., Chang C.C. (2016). Obatoclax, a Pan-BCL-2 Inhibitor, Targets Cyclin D1 for Degradation to Induce Antiproliferation in Human Colorectal Carcinoma Cells. Int. J. Mol. Sci..

[47] Schimmer A.D., O’Brien S., Kantarjian H., Brandwein J., Cheson B.D., Minden M.D., Yee K., Ravandi F., Giles F., Schuh A. (2008). A phase I study of the pan bcl-2 family inhibitor obatoclax mesylate in patients with advanced hematologic malignancies. Clin. Cancer Res..

[48] Schimmer A.D., Raza A., Carter T.H., Claxton D., Erba H., DeAngelo D.J., Tallman M.S., Goard C., Borthakur G. (2014). A multicenter phase I/II study of obatoclax mesylate administered as a 3- or 24-hour infusion in older patients with previously untreated acute myeloid leukemia. PLoS ONE.

[49] Opferman J.T., Iwasaki H., Ong C.C., Suh H., Mizuno S., Akashi K., Korsmeyer S.J. (2005). Obligate role of anti-apoptotic MCL-1 in the survival of hematopoietic stem cells. Science.

[50] Campbell C.J., Lee J.B., Levadoux-Martin M., Wynder T., Xenocostas A., Leber B., Bhatia M. (2010). The human stem cell hierarchy is defined by a functional dependence on Mcl-1 for self-renewal capacity. Blood.

[51] Steele T.M., Talbott G.C., Sam A., Tepper C.G., Ghosh P.M., Vinall R.L. (2019). Obatoclax, a BH3 Mimetic, Enhances Cisplatin-Induced Apoptosis and Decreases the Clonogenicity of Muscle Invasive Bladder Cancer Cells via Mechanisms That Involve the Inhibition of Pro-Survival Molecules as Well as Cell Cycle Regulators. Int. J. Mol. Sci..

[52] Martinez-Paniagua M.A., Baritaki S., Huerta-Yepez S., Ortiz-Navarrete V.F., Gonzalez-Bonilla C., Bonavida B., Vega M.I. (2011). Mcl-1 and YY1 inhibition and induction of DR5 by the BH3-mimetic Obatoclax (GX15-070) contribute in the sensitization of B-NHL cells to TRAIL apoptosis. Cell Cycle.

[53] Yoshino T., Shiina H., Urakami S., Kikuno N., Yoneda T., Shigeno K., Igawa M. (2006). Bcl-2 expression as a predictive marker of hormone-refractory prostate cancer treated with taxane-based chemotherapy. Clin. Cancer Res..

[54] Robertson L.E., Plunkett W., McConnell K., Keating M.J., McDonnell T.J. (1996). Bcl-2 expression in chronic lymphocytic leukemia and its correlation with the induction of apoptosis and clinical outcome. Leukemia.

[55] Calin G.A., Dumitru C.D., Shimizu M., Bichi R., Zupo S., Noch E., Aldler H., Rattan S., Keating M., Rai K. (2002). Frequent deletions and down-regulation of micro- RNA genes miR15 and miR16 at 13q14 in chronic lymphocytic leukemia. Proc. Natl. Acad. Sci. USA.

[56] Pekarsky Y., Balatti V., Croce C.M. (2018). BCL2 and miR-15/16: From gene discovery to treatment. Cell Death Differ..

[57] Allegra D., Bilan V., Garding A., Dohner H., Stilgenbauer S., Kuchenbauer F., Mertens D., Zucknick M. (2014). Defective DROSHA processing contributes to downregulation of MiR-15/-16 in chronic lymphocytic leukemia. Leukemia.

[58] Sampath D., Liu C., Vasan K., Sulda M., Puduvalli V.K., Wierda W.G., Keating M.J. (2012). Histone deacetylases mediate the silencing of miR-15a, miR-16, and miR-29b in chronic lymphocytic leukemia. Blood.

[59] Roberts A.W., Davids M.S., Pagel J.M., Kahl B.S., Puvvada S.D., Gerecitano J.F., Kipps T.J., Anderson M.A., Brown J.R., Gressick L. (2016). Targeting BCL2 with Venetoclax in Relapsed Chronic Lymphocytic Leukemia. N. Engl. J. Med..

[60] Anderson M.A., Deng J., Seymour J.F., Tam C., Kim S.Y., Fein J., Yu L., Brown J.R., Westerman D., Si E.G. (2016). The BCL2 selective inhibitor venetoclax induces rapid onset apoptosis of CLL cells in patients via a TP53-independent mechanism. Blood.

[61] Stilgenbauer S., Eichhorst B., Schetelig J., Coutre S., Seymour J.F., Munir T., Puvvada S.D., Wendtner C.M., Roberts A.W., Jurczak W. (2016). Venetoclax in relapsed or refractory chronic lymphocytic leukaemia with 17p deletion: A multicentre, open-label, phase 2 study. Lancet Oncol..

[62] Deeks E.D. (2016). Venetoclax: First Global Approval. Drugs.

[63] Stilgenbauer S., Eichhorst B., Schetelig J., Hillmen P., Seymour J.F., Coutre S., Jurczak W., Mulligan S.P., Schuh A., Assouline S. (2018). Venetoclax for Patients With Chronic Lymphocytic Leukemia With 17p Deletion: Results From the Full Population of a Phase II Pivotal Trial. J. Clin. Oncol..

[64] Seymour J.F., Ma S., Brander D.M., Choi M.Y., Barrientos J., Davids M.S., Anderson M.A., Beaven A.W., Rosen S.T., Tam C.S. (2017). Venetoclax plus rituximab in relapsed or refractory chronic lymphocytic leukaemia: A phase 1b study. Lancet Oncol..

[65] Seymour J.F., Kipps T.J., Eichhorst B., Hillmen P., D’Rozario J., Assouline S., Owen C., Gerecitano J., Robak T., De la Serna J. (2018). Venetoclax-Rituximab in Relapsed or Refractory Chronic Lymphocytic Leukemia. N. Engl. J. Med..

[66] Fischer K., Al-Sawaf O., Bahlo J., Fink A.M., Tandon M., Dixon M., Robrecht S., Warburton S., Humphrey K., Samoylova O. (2019). Venetoclax and Obinutuzumab in Patients with CLL and Coexisting Conditions. N. Engl. J. Med..

[67] Jonas B.A., Pollyea D.A. (2019). How we use venetoclax with hypomethylating agents for the treatment of newly diagnosed patients with acute myeloid leukemia. Leukemia.

[68] Chen K.T.J., Gilabert-Oriol R., Bally M.B., Leung A.W.Y. (2019). Recent Treatment Advances and the Role of Nanotechnology, Combination Products, and Immunotherapy in Changing the Therapeutic Landscape of Acute Myeloid Leukemia. Pharm. Res..

[69] Benito J.M., Godfrey L., Kojima K., Hogdal L., Wunderlich M., Geng H., Marzo I., Harutyunyan K.G., Golfman L., North P. (2015). MLL-Rearranged Acute Lymphoblastic Leukemias Activate BCL-2 through H3K79 Methylation and Are Sensitive to the BCL-2-Specific Antagonist ABT-199. Cell Rep..

[70] Chan S.M., Thomas D., Corces-Zimmerman M.R., Xavy S., Rastogi S., Hong W.J., Zhao F., Medeiros B.C., Tyvoll D.A., Majeti R. (2015). Isocitrate dehydrogenase 1 and 2 mutations induce BCL-2 dependence in acute myeloid leukemia. Nat. Med..

[71] Niu X., Wang G., Wang Y., Caldwell J.T., Edwards H., Xie C., Taub J.W., Li C., Lin H., Ge Y. (2014). Acute myeloid leukemia cells harboring MLL fusion genes or with the acute promyelocytic leukemia phenotype are sensitive to the Bcl-2-selective inhibitor ABT-199. Leukemia.

[72] Rahmani M., Nkwocha J., Hawkins E., Pei X., Parker R.E., Kmieciak M., Leverson J.D., Sampath D., Ferreira-Gonzalez A., Grant S. (2018). Cotargeting BCL-2 and PI3K Induces BAX-Dependent Mitochondrial Apoptosis in AML Cells. Cancer Res..

[73] Konopleva M., Pollyea D.A., Potluri J., Chyla B., Hogdal L., Busman T., McKeegan E., Salem A.H., Zhu M., Ricker J.L. (2016). Efficacy and Biological Correlates of Response in a Phase II Study of Venetoclax Monotherapy in Patients with Acute Myelogenous Leukemia. Cancer Discov..

[74] Pullarkat V.A., Newman E.M. (2016). BCL2 Inhibition by Venetoclax: Targeting the Achilles' Heel of the Acute Myeloid Leukemia Stem Cell?. Cancer Discov..

[75] Kantarjian H., Oki Y., Garcia-Manero G., Huang X., O’Brien S., Cortes J., Faderl S., Bueso-Ramos C., Ravandi F., Estrov Z. (2007). Results of a randomized study of 3 schedules of low-dose decitabine in higher-risk myelodysplastic syndrome and chronic myelomonocytic leukemia. Blood.

[76] DiNardo C.D., Pratz K., Pullarkat V., Jonas B.A., Arellano M., Becker P.S., Frankfurt O., Konopleva M., Wei A.H., Kantarjian H.M. (2019). Venetoclax combined with decitabine or azacitidine in treatment-naive, elderly patients with acute myeloid leukemia. Blood.

[77] Wei A.H., Strickland S.A., Hou J.Z., Fiedler W., Lin T.L., Walter R.B., Enjeti A., Tiong I.S., Savona M., Lee S. (2019). Venetoclax Combined With Low-Dose Cytarabine for Previously Untreated Patients With Acute Myeloid Leukemia: Results From a Phase Ib/II Study. J. Clin. Oncol..

[78] DiNardo C.D., Jonas B.A., Pullarkat V., Thirman M.J., Garcia J.S., Wei A.H., Konopleva M., Dohner H., Letai A., Fenaux P. (2020). Azacitidine and Venetoclax in Previously Untreated Acute Myeloid Leukemia. N. Engl. J. Med..

[79] DiNardo C.D., Pratz K.W., Letai A., Jonas B.A., Wei A.H., Thirman M., Arellano M., Frattini M.G., Kantarjian H., Popovic R. (2018). Safety and preliminary efficacy of venetoclax with decitabine or azacitidine in elderly patients with previously untreated acute myeloid leukaemia: A non-randomised, open-label, phase 1b study. Lancet Oncol..

[80] Curtis Andrew Lachowiez G.B., Loghavi S., Zeng Z., Tapan M., Kadia, Masarova L., Takahashi K., Tippett G.D., Naqvi K., Bose P. (2020). Phase Ib/II study of the IDH1-mutant inhibitor ivosidenib with the BCL2 inhibitor venetoclax +/− azacitidine in IDH1-mutated hematologic malignancies. J. Clin. Oncol..

[81] Richard-Carpentier G., DiNardo C.D. (2019). Venetoclax for the treatment of newly diagnosed acute myeloid leukemia in patients who are ineligible for intensive chemotherapy. Ther. Adv. Hematol..

[82] Davids M.S., Roberts A.W., Seymour J.F., Pagel J.M., Kahl B.S., Wierda W.G., Puvvada S., Kipps T.J., Anderson M.A., Salem A.H. (2017). Phase I First-in-Human Study of Venetoclax in Patients With Relapsed or Refractory Non-Hodgkin Lymphoma. J. Clin. Oncol..

[83] Hughes M.E., Landsburg D.J., Rubin D.J., Schuster S.J., Svoboda J., Gerson J.N., Namoglu E., Nasta S.D. (2019). Treatment of Patients With Relapsed/Refractory Non-Hodgkin Lymphoma With Venetoclax: A Single-Center Evaluation of Off-Label Use. Clin. Lymphoma Myeloma Leuk..

[84] Kumar S.K., Harrison S.J., Cavo M., de la Rubia J., Popat R., Gasparetto C., Hungria V., Salwender H., Suzuki K., Kim I. (2020). Venetoclax or placebo in combination with bortezomib and dexamethasone in patients with relapsed or refractory multiple myeloma (BELLINI): A randomised, double-blind, multicentre, phase 3 trial. Lancet Oncol..

[85] Klanova M., Andera L., Brazina J., Svadlenka J., Benesova S., Soukup J., Prukova D., Vejmelkova D., Jaksa R., Helman K. (2016). Targeting of BCL2 Family Proteins with ABT-199 and Homoharringtonine Reveals BCL2- and MCL1-Dependent Subgroups of Diffuse Large B-Cell Lymphoma. Clin. Cancer Res..

[86] Pan R., Hogdal L.J., Benito J.M., Bucci D., Han L., Borthakur G., Cortes J., DeAngelo D.J., Debose L., Mu H. (2013). Selective BCL-2 inhibition by ABT-199 causes on-target cell death in acute myeloid leukemia. Cancer Discov..

[87] Punnoose E.A., Leverson J.D., Peale F., Boghaert E.R., Belmont L.D., Tan N., Young A., Mitten M., Ingalla E., Darbonne W.C. (2016). Expression Profile of BCL-2, BCL-XL, and MCL-1 Predicts Pharmacological Response to the BCL-2 Selective Antagonist Venetoclax in Multiple Myeloma Models. Mol. Cancer Ther..

[88] Niu X., Zhao J., Ma J., Xie C., Edwards H., Wang G., Caldwell J.T., Xiang S., Zhang X., Chu R. (2016). Binding of Released Bim to Mcl-1 is a Mechanism of Intrinsic Resistance to ABT-199 which can be Overcome by Combination with Daunorubicin or Cytarabine in AML Cells. Clin. Cancer Res..

[89] O’Neill K.L., Huang K., Zhang J., Chen Y., Luo X. (2016). Inactivation of prosurvival Bcl-2 proteins activates Bax/Bak through the outer mitochondrial membrane. Genes Dev..

[90] Zhang J., Huang K., O’Neill K.L., Pang X., Luo X. (2016). Bax/Bak activation in the absence of Bid, Bim, Puma, and p53. Cell Death Dis..

[91] Campos E.D.V., Pinto R. (2019). Targeted therapy with a selective BCL-2 inhibitor in older patients with acute myeloid leukemia. Hematol. Transfus Cell Ther..

[92] DiNardo C.D., Tiong I.S., Quaglieri A., MacRaild S., Loghavi S., Brown F.C., Thijssen R., Pomilio G., Ivey A., Salmon J.M. (2020). Molecular patterns of response and treatment failure after frontline venetoclax combinations in older patients with AML. Blood.

[93] Bodo J., Zhao X., Durkin L., Souers A.J., Phillips D.C., Smith M.R., Hsi E.D. (2016). Acquired resistance to venetoclax (ABT-199) in t(14;18) positive lymphoma cells. Oncotarget.

[94] Tausch E., Close W., Dolnik A., Bloehdorn J., Chyla B., Bullinger L., Dohner H., Mertens D., Stilgenbauer S. (2019). Venetoclax resistance and acquired BCL2 mutations in chronic lymphocytic leukemia. Haematologica.

[95] Birkinshaw R.W., Gong J.N., Luo C.S., Lio D., White C.A., Anderson M.A., Blombery P., Lessene G., Majewski I.J., Thijssen R. (2019). Structures of BCL-2 in complex with venetoclax reveal the molecular basis of resistance mutations. Nat. Commun..

[96] Blombery P., Anderson M.A., Gong J.N., Thijssen R., Birkinshaw R.W., Thompson E.R., Teh C.E., Nguyen T., Xu Z., Flensburg C. (2019). Acquisition of the Recurrent Gly101Val Mutation in BCL2 Confers Resistance to Venetoclax in Patients with Progressive Chronic Lymphocytic Leukemia. Cancer Discov..

[97] Blombery P., Thompson E.R., Nguyen T., Birkinshaw R.W., Gong J.N., Chen X., McBean M., Thijssen R., Conway T., Anderson M.A. (2020). Multiple BCL2 mutations cooccurring with Gly101Val emerge in chronic lymphocytic leukemia progression on venetoclax. Blood.

[98] Correia C., Schneider P.A., Dai H., Dogan A., Maurer M.J., Church A.K., Novak A.J., Feldman A.L., Wu X., Ding H. (2015). BCL2 mutations are associated with increased risk of transformation and shortened survival in follicular lymphoma. Blood.

[99] Davies A.J., Rosenwald A., Wright G., Lee A., Last K.W., Weisenburger D.D., Chan W.C., Delabie J., Braziel R.M., Campo E. (2007). Transformation of follicular lymphoma to diffuse large B-cell lymphoma proceeds by distinct oncogenic mechanisms. Br. J. Haematol..

[100] Fresquet V., Rieger M., Carolis C., Garcia-Barchino M.J., Martinez-Climent J.A. (2014). Acquired mutations in BCL2 family proteins conferring resistance to the BH3 mimetic ABT-199 in lymphoma. Blood.

[101] Caenepeel S., Brown S.P., Belmontes B., Moody G., Keegan K.S., Chui D., Whittington D.A., Huang X., Poppe L., Cheng A.C. (2018). AMG 176, a Selective MCL1 Inhibitor, Is Effective in Hematologic Cancer Models Alone and in Combination with Established Therapies. Cancer Discov..

[102] Yi X., Sarkar A., Kismali G., Aslan B., Ayres M., Iles L.R., Keating M.J., Wierda W.G., Long J.P., Bertilaccio M.T.S. (2020). AMG-176, an Mcl-1 Antagonist, Shows Preclinical Efficacy in Chronic Lymphocytic Leukemia. Clin. Cancer Res..

[103] Wei A.H., Roberts A.W., Spencer A., Rosenberg A.S., Siegel D., Walter R.B., Caenepeel S., Hughes P., McIver Z., Mezzi K. (2020). Targeting MCL-1 in hematologic malignancies: Rationale and progress. Blood Rev..

[104] Reyna D.E., Garner T.P., Lopez A., Kopp F., Choudhary G.S., Sridharan A., Narayanagari S.R., Mitchell K., Dong B., Bartholdy B.A. (2017). Direct Activation of BAX by BTSA1 Overcomes Apoptosis Resistance in Acute Myeloid Leukemia. Cancer Cell.

[105] Stornaiuolo M., La Regina G., Passacantilli S., Grassia G., Coluccia A., La Pietra V., Giustiniano M., Cassese H., Di Maro S., Brancaccio D. (2015). Structure-based lead optimization and biological evaluation of BAX direct activators as novel potential anticancer agents. J. Med. Chem..

[106] Liu Z., Ding Y., Ye N., Wild C., Chen H., Zhou J. (2016). Direct Activation of Bax Protein for Cancer Therapy. Med. Res. Rev..

[107] Casara P., Davidson J., Claperon A., Le Toumelin-Braizat G., Vogler M., Bruno A., Chanrion M., Lysiak-Auvity G., Le Diguarher T., Starck J.B. (2018). S55746 is a novel orally active BCL-2 selective and potent inhibitor that impairs hematological tumor growth. Oncotarget.

